# Stress genomics revisited: gene co-expression analysis identifies molecular signatures associated with childhood adversity

**DOI:** 10.1038/s41398-020-0730-0

**Published:** 2020-01-27

**Authors:** Linda Dieckmann, Steve Cole, Robert Kumsta

**Affiliations:** 1 Department of Genetic Psychology, Faculty of Psychology, Ruhr University Bochum, Bochum, Germany; 2 Division of Hematology/Oncology, David Geffen School of Medicine, University of California, Los Angeles, CA USA; 3 Cousins Center for Psychoneuroimmunology, University of California, Los Angeles, CA USA; 4 Department of Psychiatry and Biobehavioral Sciences, University of California, Los Angeles, CA USA

**Keywords:** Diagnostic markers, Genomics

## Abstract

Childhood adversity is related to an increased risk for psychopathology in adulthood. Altered regulation of stress response systems, as well as the changes in stress-immune interplay have been suggested as potential mechanisms underlying these long-term effects. We have previously shown altered transcriptional responses to acute psychosocial stress in adults reporting the experience of childhood adversity. Here, we extend these analyses using a network approach. We performed a co-expression network analysis of genome-wide mRNA data derived from isolated monocytes, sampled 3 h after stress exposure from healthy adults, who experienced childhood adversity and a matched control group without adverse childhood experiences. Thirteen co-expression modules were identified, of which four modules were enriched for genes related to immune system function. Gene set enrichment analysis showed differential module activity between the early adversity and control group. In line with previous findings reporting a pro-inflammatory bias following childhood adversity, one module included genes associated with pro-inflammatory function (hub genes: *IL6, TM4SF1, ADAMTS4, CYR61, CCDC3*), more strongly expressed in the early adversity group. Another module downregulated in the early adversity group was related to platelet activation and wound healing (hub genes: *GP9, CMTM5, TUBB1, GNG11, PF4)*, and resembled a co-expression module previously found over-expressed in post-traumatic stress disorder resilient soldiers. These discovery analysis results provide a system wide and more holistic understanding of gene expression programs associated with childhood adversity. Furthermore, identified hub genes can be used in directed hypothesis testing in future studies.

## Introduction

The experience of adversity and traumatic events in early life are consistently identified as risk factors for the development of a range of mental and physical disorders^1–4^. Over the past years, research aimed at uncovering the mechanisms linking early adversity, and disease risk has intensified and is considered a major health care priority. In particular, regulation of the stress response, the immune response, and the interplay between the two systems have come into focus: a number of studies have shown that inflammatory responses and the functioning of the hypothalamic-pituitary-adrenal (HPA) axis constitute pathways through which childhood adversity may lead to disorder manifestation^5–9^.

Adverse experiences in early life seem to shift the innate immune response toward a more pro-inflammatory state, and this pro-inflammatory bias continues to be observed into adulthood^10–12^. Furthermore, altered adrenocorticotropic hormone (ACTH) and/or cortisol reactivity has been observed in children and adults with adverse childhood experiences in prospective^13–15^ and retrospective studies^16–19^. These long-term effects speak toward a biological embedding of experience, and it has been suggested that early adversity leads to a programming of molecular systems and related gene expression profiles, resulting in an altered stress response and in differences in the sensitivity of immune genes toward stress signals^20–23^.

There is evidence of extensive bidirectional interplay between mediators of the stress response and immune system effectors^24–26^, and growing evidence of social regulation of gene expression programs in innate immune cells^27^. Activation of the HPA axis inhibits both antiviral and pro-inflammatory gene modules, and activation of the sympathetic nervous system only inhibits antiviral responses and stimulates pro-inflammatory genes^26^. It was shown that diverse forms of social adversity, including low socioeconomic status, chronic stress, and post-traumatic stress disorder (PTSD), evoke a conserved transcriptional response characterized by decreased expression of antiviral response genes and increased expression of pro-inflammatory genes^27^. From an evolutionary perspective, such an anticipation of challenging situations manifested in shifts to the basal leukocyte transcriptome could constitute a survival advantage in hostile environments, by preparing an organism to respond to injury in fight-or-flight situations^22,26^. On the other hand, this response pattern can become maladaptive in modern times, where daily stressors are mainly of psychosocial nature and occur without injury.

Taken together, exposure to different types of unfavorable life circumstances is associated with distinct transcriptional profiles in leukocytes, with monocytes as the most transcriptionally sensitive subtype for social conditions and traumatic experiences^28–31^. However, the majority of studies focused on exposure to current adversity, and investigated basal gene expression profiles. Given the regulatory function of stress system activation on immune gene expression, and given increased levels of pro-inflammatory cytokines in adults reporting childhood adversity^10,32^, we previously investigated whether a pro-inflammatory bias might be observed in response to acute stress exposure on the transcriptional level. Examination of genome-wide mRNA expression changes in monocytes following acute stress exposure in healthy adults with and without early trauma^33^ revealed several stress-responsive transcripts, as well as transcripts with differential expression between the groups. Furthermore, transcription factor binding motif analysis showed an increased activity of pro-inflammatory upstream signaling in the early adversity group. Results of this provided first evidence for persistent alterations in transcriptional control of stress-responsive immune cells associated with the experience of childhood adversity.

Here, we expand upon these findings through a hypothesis-free discovery analysis of empirical differences in gene co-expression patterns. The organization of gene expression data into networks has been argued to provide a robust and reproducible structure, and may have significant functional implications that cannot be derived from standard differential expression analysis at the level of individual genes^34,35^. In the past, it was shown that co-expressed genes are often involved in the same or related biological pathways^36,37^, and can be informative of the biological state of an individual^38^. As a system-based approach, co-expression analysis places each gene in the context of its molecular system and takes into account interactions between the components^39–41^. At the same time, it allows to integrate multiple levels of data, therewith reduces dimensionality and the multiple testing problem.

The aim of this analysis was to identify stress-related molecular signatures of early adversity, using an analytic approach that is promising to complement single gene analyses with additional insights. The focus was set on expression differences after acute stress exposure. By constructing an unsupervised gene co-expression network from samples taken from adults with and without childhood adversity, we expected to (i) identify differences in the activity of various co-expression modules; and (ii) to identify broader functional signatures related to pro-inflammatory signaling compared to the previous single gene analysis.

## Materials and methods

### Sample

Data used in this paper were generated in a project investigating the long-term consequences of childhood adversity on hormonal and genomic responses to stress^33^ and emotion recognition abilities^42^. For the current publication, gene expression data were reanalyzed with regard to gene co-expression. For details on study procedure, sample characteristics, and additionally collected data, see Schwaiger et al.^33,42^. In brief, the study included 60 healthy adults (40 males and 20 females). Our sample was adequately powered for the gene co-expression analysis, as a minimum of 15–20 samples are recommended for weighted gene co-expression networks^43^. All participants were free of mental disorders for the past 12 months, screened for with the German version of the Structured Clinical Interview for DSM Disorders (SKID I & II)^44^. Further exclusion criteria were the intake of psychoactive or cortisol-containing medication, and the use of oral contraceptives for females. The German 28-item version of the Childhood Trauma Questionnaire (CTQ)^45,46^ was used to assess the presence of childhood trauma (sexual, physical and emotional abuse, and physical and emotional neglect). CTQ cutoff scores for moderate to severe exposure to traumatic events were used to classify subjects as positive for a history of childhood adversity (*N* = 30, mean age 52.57 years with SD = 5.52 years). The group assignment was validated in a structured interview with the Early Trauma Inventory^47,48^. The participants in the control group scored below cutoff on all CTQ subscales (*N* = 30, mean age 51.47 years with SD = 4.64 years), and were matched to the early adversity group for gender, age, current, and childhood socio-economic status (SES). The study was approved by the Ethics Committee of the Albert-Ludwigs-University Freiburg (183/11). All participants gave informed consent and were paid 100 Euro for participation.

### Experimental procedures

Participants were exposed to the Trier Social Stress Test (TSST)^49^, a standardized 15-min laboratory stress protocol. TSST panel members were blinded to group status. For RNA extraction, 10 ml ethylenediaminetetraacetic acid blood samples were collected at 45 min before, and 45 min and 180 min after the TSST. Immunomagnetic cell separation (MACS; Miltenyi Biotec, Germany) was used for isolation of CD14^+^ monocytes. Purity of the isolated monocyte population was checked with fluorescence-activated cell sorting analyses and showed high purity values (mean = 92.92%, SE = 0.59). Isolated cells were resuspended in lysis buffer RA1, shock-frozen in liquid nitrogen, and stored at −80 °C. RNA was extracted (Macherey-Nagel, Germany) and RNA integrity number values ranged from 8.0 to 10.0 (mean = 9.7, SE = 0.03), which were assessed with the Agilent 2100 bioanalyzer (Agilent Technologies). For profiling on Agilent Whole Human Genome Oligo Microarrays 8 × 60 K V2, 100 ng of RNA was used. To avoid batch effects, all samples were randomized within and between arrays. The assays were performed with the manufacturer´s standard protocol at the Molecular Service Center (Miltenyi Biotech).

### Data preprocessing

Quantile-normalized gene expression values were log2-transformed with the R (version 3.1.1) package limma^50^. From a total of 50,683 transcripts, multiple transcripts corresponding to one gene were identified. Mean expression values over all samples included in the network construction were calculated for every transcript. Only the transcript with highest mean expression was kept for every gene associated with multiple transcripts, leading to 32,080 unique genes which were used in the further analyses.

### Gene co-expression network analysis

For the construction of a weighted and signed co-expression network, we used the R (version 3.5.1) package CEMiTool^51^ (version 1.9.3). This offers an implemented unsupervised gene filtering method and more automated parameter selection than the widely used package WGCNA^43^, aiming to enhance the reproducibility of results. To reduce noise, the filtering procedure starts with removing the 25% genes with lowest mean expression. Then, the variance of genes is modeled as an inverse gamma distribution, and genes are filtered based on a *p*-value. Here, we chose the standard *p*-value of 0.1, as this was shown to be a good compromise between noise reduction and information loss^51^. After filtering, 3612 genes remained in the analysis. As the mean-variance dependency was low in the data (*R*² = .061), we applied no variance stabilization transformation. To construct the network, Pearson correlation was calculated as a similarity measure for all pair-wise genes. To preserve the sign of correlation, the absolute value of correlation was transformed, and scaled into the [0,1] interval^52^. Then, soft thresholding of the correlation matrix was used to determine the connection strengths and preserve the continuous nature of the gene co-expression information. The similarity values were raised to a power of *β* (soft power adjacency function), leading to the weighted adjacency matrix^52^. In CEMiTool, the *β* parameter is selected by an algorithm, which is based on the concept of Cauchy sequences. For adherence to the scale-free topology, only *β* values with *R*² > 0.80 are considered, while lower *β* values are preferred due to considerations of network connectivity. The default parameters in CEMiTool were contained to preserve the advantages of more reliable and consistent results with the automatic selection of *β*. This led to a power of *β* = 5 (scale-free *R*² = .834). From the adjacency matrix, the topological overlap measure (TOM) can be calculated, representing shared neighborhood between genes^53^. Afterward, subtracting the TOM from 1 leads to a topology dissimilarity measure, which can be used as input for the clustering procedure. Clusters of highly co-expressed genes (modules) were detected by a dynamic algorithm for selecting branches of the hierarchical clustering dendrogram implemented in the Dynamic Tree Cut package^54^. The minimum number of genes per submodule was set to the default of 30 genes and the module merging correlation threshold for eigengene similarity was 0.8. To determine biological functions associated with the modules, the C5 Gene Ontology (GO) gene set list from MSigDB^55^ was used to perform an over representation analysis via the clusterProfiler R package^56^ in CEMiTool. Top gene sets enriched on each co-expression module were detected by the hypergeometric test with *p* = 0.05 and Benjamini–Hochberg correction for multiple testing. To visualize interactions between the genes in each co-expression module, the combined human interaction data from GeneMania^57^ was included in the analysis. In order to assess differences in module activity between the early adversity and control group, a gene set enrichment analysis with the fgsea R package^58^ was performed with the default values in CEMiTool. Here, genes from co-expression modules are used as gene sets and the *z*-score normalized expression of the samples within each group are ranked in the analysis. The enrichment score (ES) is normalized by taking into account the size of each gene set, leading to a normalized ES (NES). The proportion of false positives is controlled by calculating the false discovery rate corresponding to each NES and an adjustment of the respective *p*-value. Ultimately, the five most highly connected genes (Hub genes)^52^ were determined for each module. R Code is available on request.

## Results

We constructed a weighted gene co-expression network for peripheral blood monocyte RNA samples taken from healthy adults, with and without adverse childhood experiences 180 min after an acute psychosocial stress exposure. The network comprised 13 modules, with sizes ranging from 99 to 772 genes (Supplementary Table 1). No genes were assigned as not correlated.

**Fig. 1:**
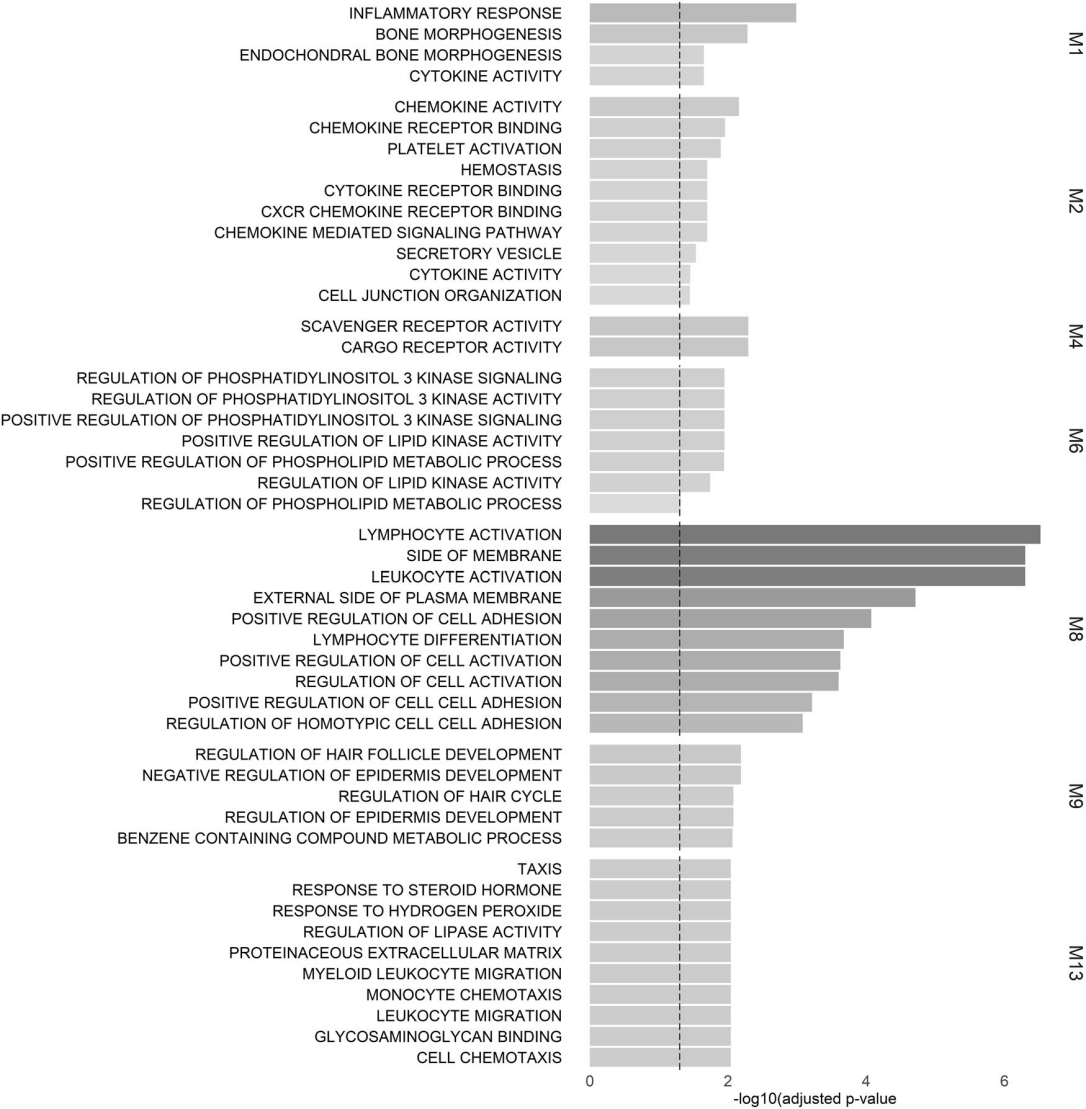
Over representation analysis for modules identified to be significantly enriched for functional annotation. Bar graphs show the −log_10_ adjusted *p*-value of the enrichment between genes in modules and GO gene sets from MSigDB, the vertical dashed line indicates an adjusted *p*-value of 0.05. The color intensities of the bars are proportional to the adjusted *p*-value. Only significant GO gene sets are shown for each module.

Overall, seven modules were found to have at least one significantly enriched pathway in the over representation analysis (Fig. 1; see Supplementary Table 2 for comprehensive statistics).

Following our hypothesis of altered stress-immune interplay, modules M1, M2, M8, and M13 are of particular interest, as they are all related to the immune system.

For characterization of putative functions of genes clustering in modules, it can be useful to examine the role of some of its most central genes. While hub genes in general are crucial for the network’s structure^59^, intramodular hub genes are often of clinical importance and biological relevance^60^.

**Fig. 2:**
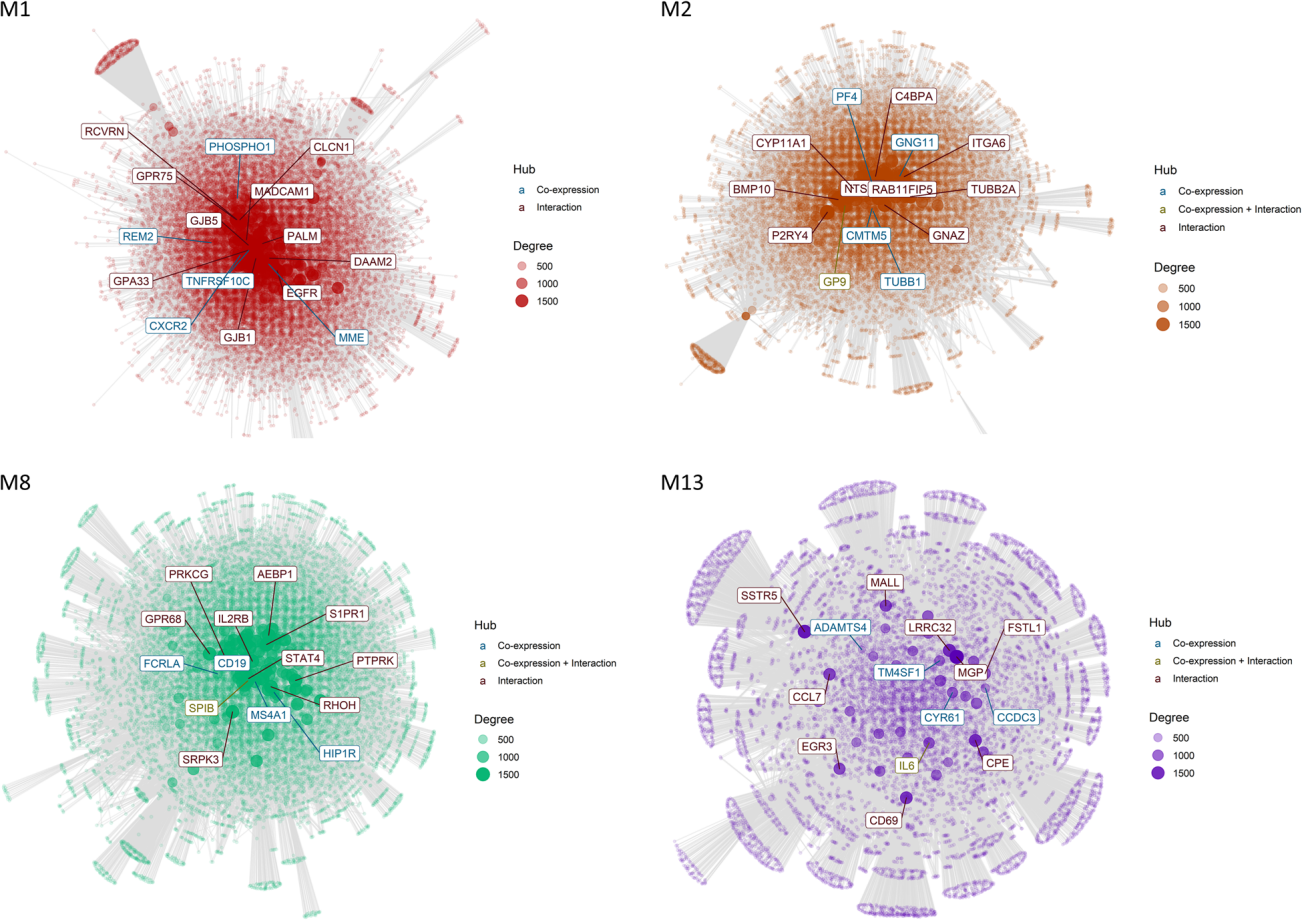
Module gene networks. Interactions between genes in the co-expression module are visualized and genes are colored based on their source. If they are derived from the interaction file they are colored red, if they are module hubs they are colored blue, and in case both is true they are colored green. The degree (connection strength) is reflected by the node’s sizes.

Module 13, relatively over-expressed in the early adversity group, contains transcripts enriched for leukocyte migration, monocyte chemotaxis, and response to steroid hormones. A central hub gene, also obtained from the interaction data, is *IL6* (Fig. 2). It codes for a cytokine that is primarily produced at sites of acute and chronic inflammation, and implicated in the development of various autoimmune and chronic inflammatory diseases^61^. Another hub of M13 is *CYR61*, responsible for a matricellular protein that was identified to be important for inflammation and tissue repair in adulthood, and related to chronic inflammation^62,63^. Three more genes with high intramodular activity were identified through interaction analysis, all with known pro-inflammatory function: *EGR3*, a classical pro-inflammatory transcription factor^64^, *CD69*, a well-known T-cell activation marker^65^, and *CCL7*, a monocyte chemotaxis chemokine^66^.

The largest module, M1, consists of genes that are over-proportionally related to inflammatory responses. Central genes of M1 can be seen in the module’s network graph (Fig. 2). Among the hub genes of M1 are for example *TNFRSF10C* and *CXCR2*. *TNFRSF10C* codes for a protein that is a member of the tumor necrosis factor (TNF) receptor superfamily and one of several TRAIL (TNF-related apoptosis-inducing ligand-like) decoy receptors. *CXCR2* codes for the interleukin 8 receptor, fulfills complex regulatory functions in the innate immune system and was defined as a potential target for the therapeutic treatment of inflammatory processes^67,68^.

Another module, M2, is related to chemokine and platelet activation. Chemokines are involved in the migration of leukocytes and are both central for pro-inflammatory responses and homeostasis of the immune system^69^. They can also be activators of platelets, which are themselves important for hemostasis and host defense^70^. These processes are again reflected in the module’s central nodes (Fig. 2). Gene *GP9* codes for a membrane glycoprotein on the surface of human platelets, and is both implicated by co-expression and inferred gene interaction data. The intramodular hub gene *TUBB1* is coding for a member of the beta tubulin protein family, and gene *PF4* encodes a member of the CXC chemokine family that was found to initiate a signal transduction cascade of acute and delayed functions, including phagocytosis, respiratory burst, survival, and the secretion of cytokines^71^.

Module 8 contains transcripts related to lymphocyte activation. A central hub gene of M8 is *CD19* (Fig. 2), which is coding for a cell surface molecule assembling the antigen receptor of B lymphocytes, decreasing the threshold for antigen receptor-dependent stimulation. The intramodular hub gene *MS4A1* also encodes a B-lymphocyte surface molecule playing a role in the development of B Cells.

**Fig. 3:**
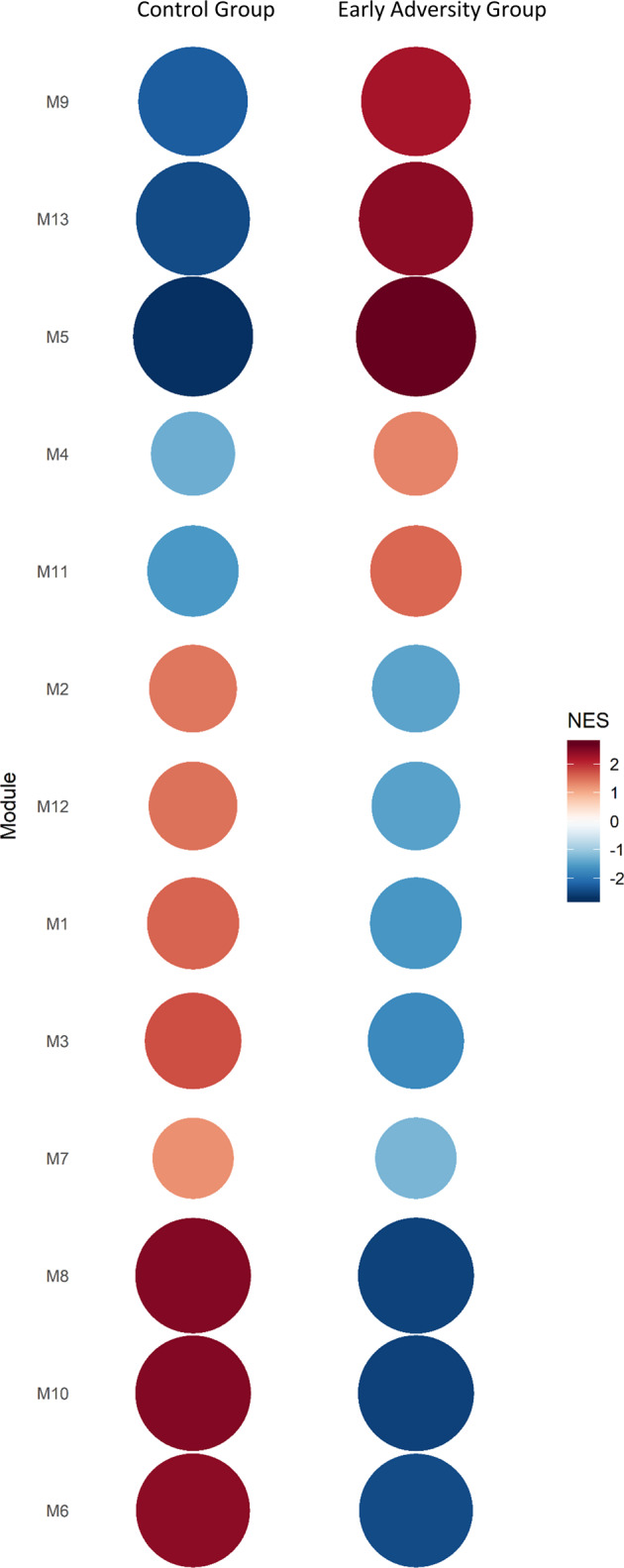
Gene set enrichment analysis showing the module activity by group. The circle and color intensities are proportional to the NES values, red and blue show respectively higher and lower module activity.

All identified modules showed differential activity between the early adversity and control group (Fig. 3; see Supplementary Table 3 for a complete listing of NES and adjusted *p*-values). While the activity of modules M1, M2, and M8 is relatively lower in the early adversity group, genes of M13 are relatively upregulated in the early adversity group.

**Fig. 4:**
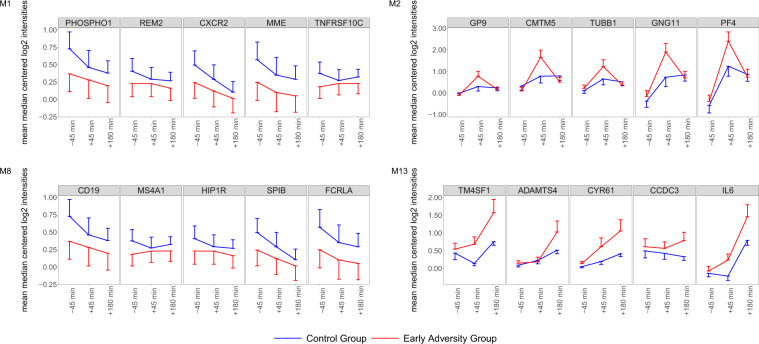
Expression levels of the top five intramodular hub genes over time. Gene expression levels were determined for probes collected 45 min before stress exposure, as well as 45 and 180 min after stress exposure. Error bars represent one standard error of the mean.

In order to get an impression of expression patterns over time for these modules, we illustrated the expression of the top five intramodular hub genes across the three time points (45 min pre, and 45 and 180 min post-stress; Fig. 4). It can be seen that the strength, but not the direction of group differences in expression levels changes over time for modules M1, M8, and M13. On the other hand, hub genes of M2 shift also in their direction of group differences between the time points. Overall, these results indicate altered interactions between the stress and immune system in adults with a history of childhood adversity, reflected in differential activity patterns of four co-expression modules enriched for genes with immune-related functions after acute stress exposure.

## Discussion

In the present study, we aimed to identify stress-related transcriptional signatures of early life adversity on a system level. By constructing a gene co-expression network from data collected after acute psychosocial stress exposure, we identified several modules consisting of genes whose coordinated functions are potentially crucial for an adaptive stress response. We found four modules to be enriched for genes involved in functions of the immune system, which is consistent with evidence for a close relationship between acute psychological stress and regulatory immune system activities^25^. Additionally, these modules showed different expression patterns between adults with and without childhood adversity. This may be indicative of dysregulations in the stress-immune axis, assumed to be a consequence of early life stress^72,73^. In general, a key question in the study of how stress and other types of adversities increase disease risk concerns the mechanisms of how psychosocial processes are transduced to the molecular level. The investigation of gene expression signatures has emerged as a promising way for capturing molecular manifestations of both acute and chronic stress. For instance, a series of investigations has demonstrated that various socio-environmental risk factors, including poverty, bereavement, and chronic stress, were associated with specific gene expression patterns, characterized by upregulated transcripts involved in inflammation, and downregulated transcripts involved in antiviral responses (termed conserved transcriptional response to adversity; CTRA). In the face of stress exposure, this transcriptional program is thought to promote chronic low-grade inflammation, and thus provides a mechanistic link between stress and the development of inflammation-related diseases^74^. Whereas studies investigating gene expression pattern using the CTRA framework focus on specific gene sets and identification of upstream transcription factors, we used gene co-expression analysis, which focusses on interactions between genes placed in the context of molecular systems. Results presented here provide converging evidence for a programming of immune gene responses to stress following early adversity, as we found two modules involved in inflammation, and one module involved in wound healing with differential activity between groups.

The first module of note is a monocyte activation module (M13) that contains pro-inflammatory genes with *IL6* as a hub gene, and which is more active in the early adversity group. Notably, *IL6* was also found to be significantly upregulated from pre-stress to 180 min post-stress in our previous analysis with standard differential gene expression analysis, but no interaction was found with the group assignment^33^. This illustrates how considering not only a single gene, but also interactions among genes can complement our insights into molecular processes associated with a complex phenotype. In general, this result is in accordance with previous studies showing inflammatory activities after acute psychosocial stress and exaggerated inflammation in individuals with early life stress^10,32,75^. While an increase in peripheral inflammation after acute stress can function as an anticipatory protection against injury and infection, stable differences toward higher inflammatory responses to daily stressors can become maladaptive after repeated or chronic psychological stress, especially if not carefully regulated and terminated^76^. In turn, a state of low-grade chronic inflammation increases the vulnerability for a range of mental and physical disorders^77,78^. There is growing evidence for a causal link between inflammatory markers and depression, emphasizing the potential use of anti-inflammatory drugs in prevention and treatment of this disorder^79–81^. In addition, Tawakol et al.^82^ found in a prospective study that the association between stress-associated amygdalar activity and cardiovascular disease was mediated through arterial inflammation, and Khandaker et al.^80^ report that the comorbidity between coronary heart disease and depression arises from shared environmental factors. Together, these findings support the hypothesis that stress-associated inflammatory activities constitute a shared etiologic factor for distinct multifactorial diseases. The differences in module 13 activity observed here thus support the overall picture of a pro-inflammatory bias in individuals reporting early life adversity, and provide further clues as to why early psychosocial stress is not only related to increased risk for mental health problems, but also for inflammation-related cardiovascular and metabolic diseases.

A further noteworthy module is M2, enriched for genes associated with chemokine and platelet activation, important for homeostasis of the immune system and wound healing processes. Whereas this module can be considered a platelet activation module, we found all hub genes upregulated 45 min post-stress exposure in monocytes^33^, with relatively higher expression increases in the early adversity group. Gene set enrichment analysis found lower module activity 180 min post-stress in the early adversity group, which is not reflected in the expression of the hub genes at this time point (Fig. 4). However, gene set enrichment analysis takes into account all genes in the network, and it is presumably other members of the network that account for the overall reduction in module activity at that time point. Additional time points and further dissection of this module into distinct subcomponents might clarify whether there are differences in the temporal dynamics of this gene expression program associated with early adversity, with relatively earlier and stronger activity, and subsequent relative downregulation. Furthermore, it is unclear whether the observed module activity reflects gene expression signatures in platelets (which have little intrinsic transcriptional ability), or whether genes of this co-expression module also have distinct or complementary functions within monocytes (e.g., representing a monocyte transcriptional response to platelet activation). Activation of coagulation after acute psychosocial stress can be found in healthy subjects and is thought of as an adaptive physiological response, but a hypercoagulable state might be an indicator of imbalances to this hemostatic system and linked to disease^83,84^. Of note, a co-expression module involved in hemostasis and platelet activation which resembles our module was identified to be over-expressed in PTSD resilient military personnel after deployment^85^. Direct comparison of the two studies is, however, difficult, as Breen et al. found differences in unstimulated gene expression between soldiers with and without PTSD after recent trauma exposure, and we see the strongest differences following acute stress exposure in healthy individuals, reporting the experience of childhood adversity. The evidence of differences in module 2 activity does suggest, however, that regulation of wound healing processes might potentially be affected by early adversity.

Module 1 is composed of genes annotated as involved in inflammatory responses and bone morphogenesis, and relatively downregulated in the early adversity group. At first glance, this seems to be somewhat contradictory to studies reporting elevated pro-inflammatory tendencies following childhood adversity^10,11^. Here, the temporal dynamics of the five hub genes shows that these transcripts are downregulated from pre- to 180 min post-stress in both groups, so that this module might represent a subset of genes related to inflammation negatively regulated by stress. It also is possible that the downregulation observed here reflects other aspects of these genes’ pleiotropic function rather than their specific role in inflammation.

Module 8 contains CD19 as a hub gene, a definitive marker of B lymphocytes, so it can be considered a B cell activation module. Although purity of our isolated cell pool was very high, with 93% monocytes^33^, further analyses of cellular heterogeneity based on DNA methylation profiles showed that B lymphocytes were present, at levels of 2–5% (Supplemental Fig. 1). It remains unclear whether module 8 reflects a B-cell-specific co-expression module resulting from low-level B cell contamination or whether the module contains transcripts that are also expressed to some extent in monocytes. In general, caution must be exercised in interpreting co-expressed gene sets based on annotated biological functions through indirect bioinformatic inference, e.g., over representation analysis.

Further limitations need mention. We decided to construct the co-expression network from samples collected 3 h after the psychosocial stress exposure. This is a time point where transcriptional effects of the TSST can be expected, and we avoided dependencies in the network structure that might have been induced by including several probes from the same individual. Nevertheless, other sampling times and a longer follow-up after the stress exposure could have been also informative. Because the present analysis did not focus on change from baseline, and derived co-expression modules from a single post-stress time point, it cannot be determined which effects observed here represent stable individual differences that would also appear under basal conditions and which represent effects that occur only in response to stress. It is also not clear if the structure and composition of the co-expression modules identified here is stable or would be different under basal conditions (or in response to other stimuli or stressors). In addition, childhood adversity was assessed with a retrospective measure, and a more sensitive classification of type, time point and duration of childhood trauma might reveal further differences. This study isolated monocytes for analysis, and any effects of early adversity that manifest through other cell types (e.g., T cells, B cells, NK cells, etc.), or changes in the relative prevalence of monocytes compared to these other cell types, are missed in this analysis. Also, the sample size was limited due to the experimental procedure, and while the groups were matched for gender, age, current, and childhood SES, there remain some potential sources of variance not accounted for, like minor hidden infections, behavioral differences, possible differences in substance use, eating behavior (and related BMI), and other health behaviors. Linked to this, it cannot be ruled out that some of the group differences may have been driven by a slight imbalance of a small number of individuals with exceptionally high gene expression values. Lastly, there was an uneven sex distribution in our sample, with twice as many females compared to men taking part in the study. Given the specific statistical analysis employed, the present study was not able to address whether sex differences might exist in the magnitude of the reported effects, or whether the adversity-related differences might be confounded by the uneven sex distribution. These questions represent important topics for future research.

In summary, the results of our study reveal groups of genes that are co-expressed after an acute psychosocial stress exposure, and which probably constitute functional molecular systems related to the acute stress response. We highlight four modules involved in immune system-related functions, possibly reflecting the interplay between the stress and immune system on a transcriptional level. Importantly, these modules also show different activity between our groups. Therefore, they constitute potential targets to better understand the effects of adverse childhood experiences on stress-related gene expression programs. Furthermore, the network approach provides a more integral view on the downstream pathways of stress, and possible dysregulations in this system. These insights are a step toward a more comprehensive understanding of how childhood adversity increases the risk for a range of somatic and psychiatric disorders.

### GEO accession

The data discussed in this publication have been deposited in NCBI’s Gene Expression Omnibus and are accessible through GEO Series accession number GSE70603 (http:// www.ncbi.nlm.nih.gov/geo/query/acc.cgi?acc=GSE70603).

## Supplementary information

Supplemental Figure 1

Supplemental Figure 1 Legend

Supplemental Tables
