# Supplementary figures and images for: Stress genomics revisited: gene co-expression analysis identifies molecular signatures associated with childhood adversity
Source: Transl Psychiatry. 2020 Jan 27;10:34. doi: 10.1038/s41398-020-0730-0 (PMC7026041; doi:10.1038/s41398-020-0730-0)

Sample\_ID

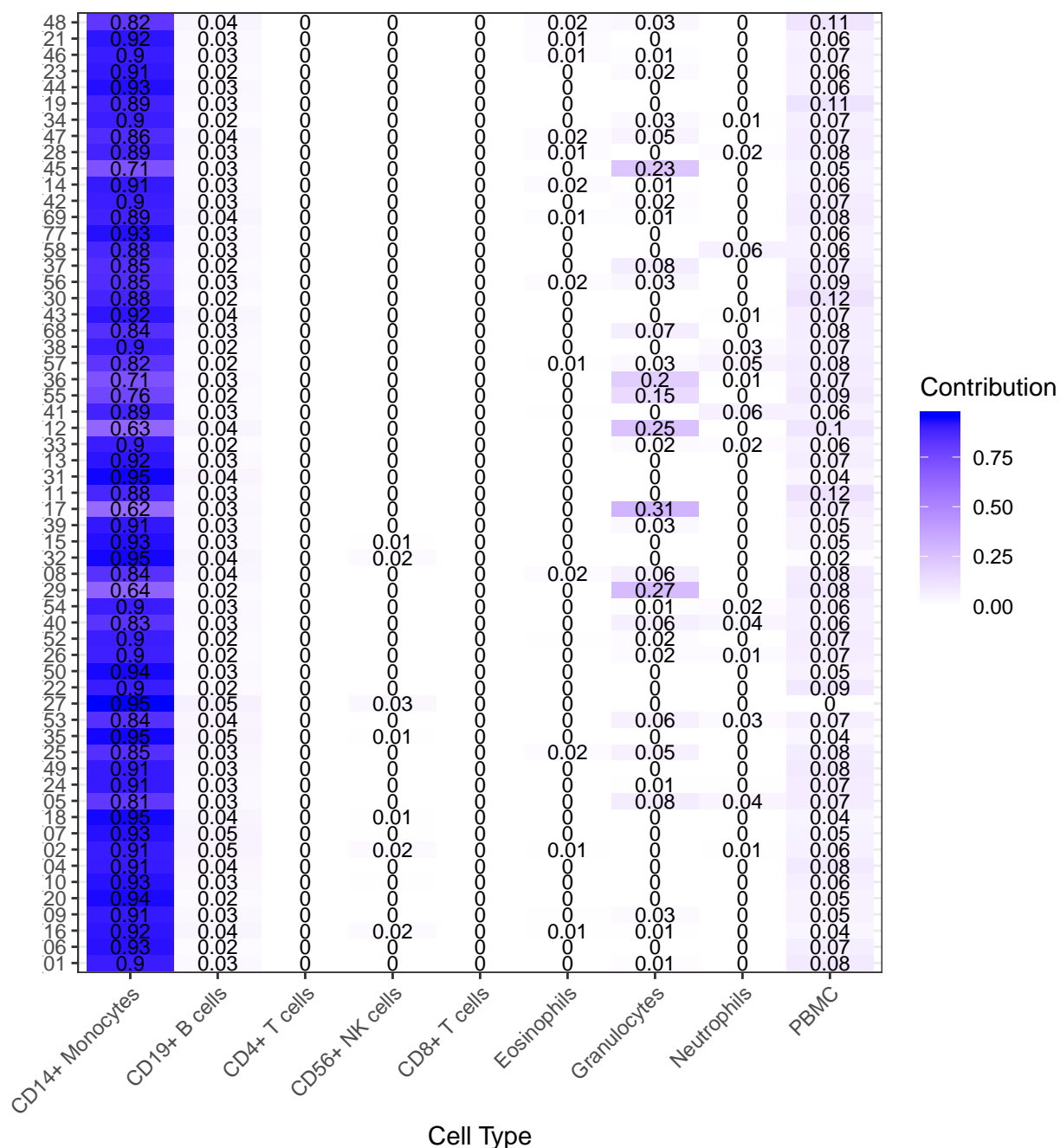

Supplement: Supplementary file 1 — Supplemental Figure 1 [file 41398_2020_730_MOESM1_ESM.pdf]
