# Supplementary material for: Stress genomics revisited: gene co-expression analysis identifies molecular signatures associated with childhood adversity
Source: Transl Psychiatry. 2020 Jan 27;10:34. doi: 10.1038/s41398-020-0730-0 (PMC7026041; doi:10.1038/s41398-020-0730-0)
Supplement: Supplementary file 2 — Supplemental Figure 1 Legend [file 41398_2020_730_MOESM2_ESM.docx]

**Supplemental Figure 1**: Cell type composition of the investigated blood samples estimated with the RnBeads bioinformatics pipeline, using Illumina 450k array DNA methylation data.
