# Supplementary material for: Stress genomics revisited: gene co-expression analysis identifies molecular signatures associated with childhood adversity
Source: Transl Psychiatry. 2020 Jan 27;10:34. doi: 10.1038/s41398-020-0730-0 (PMC7026041; doi:10.1038/s41398-020-0730-0)
Supplement: Supplementary file 3 — Supplemental Tables [file 41398_2020_730_MOESM3_ESM.docx]

# Supplementary Tables

**Supplementary Table 1. Identified Modules with corresponding number of genes and top five hub genes**

| Module | number of genes | | hub genes |
| --- | --- | --- | --- |
| M1 | | 772 | PHOSPHO1, REM2, CXCR2, MME, TNFRSF10C |
| M2 | | 429 | GP9, CMTM5, TUBB1, GNG11, PF4 |
| M3 | | 414 | XLOC_004693, XLOC_000056, KCNJ18, LOC100507412, LOC645586 |
| M4 | | 409 | MMRN2, XLOC_012768, XLOC_006388, XLOC_012621, FRMD1 |
| M5 | | 355 | lincROR, XLOC_002948, XLOC_l2_007097, C1orf61, WNT10B |
| M6 | | 325 | SUV39H1, XLOC_008357, XLOC_006936, RALGAPA1, XLOC_006297 |
| M7 | | 282 | KDM5D, RPS4Y1, RPS4Y2, LOC100509121, UTY |
| M8 | | 213 | CD19, MS4A1, HIP1R, SPIB, FCRLA |
| M9 | | 127 | LOC441052, XLOC_002852, XLOC_003202, XLOC_009714, GPR123 |
| M10 | | 99 | XLOC_009585, XLOC_012535, XLOC_008252, XLOC_013592, ADH4 |
| M11 | | 89 | LOC100507000, XAGE2B, TSPYL6, XLOC_000745, ZNF556 |
| M12 | | 50 | TRMT61A, XLOC_l2_005415, XLOC_l2_002894, RAB36, XLOC_000681 |
| M13 | | 48 | TM4SF1, ADAMTS4, CYR61, CCDC3, IL6 |

**Supplementary Table 2. Comprehensive statistics of the over representation analysis for modules identified to be significantly enriched for functional annotation**

| Module | Gene Ontology Term^1^ | Count | GeneRatio | BgRatio | adjusted^2^  p-value | |
| --- | --- | --- | --- | --- | --- | --- |
| M1 | Inflammatory Response | 27 | 27/336 | 455/17107 |  | 0.001 |
|  | Bone Morphogenesis | 10 | 10/336 | 80/17107 |  | 0.005 |
|  | Cytokine Activity | 15 | 15/336 | 220/17107 |  | 0.022 |
|  | Endochondral Bone Morphogenesis | 07 | 07/336 | 46/17107 |  | 0.022 |
| M2 | Chemokine Activity | 07 | 07/217 | 49/17107 |  | 0.007 |
|  | Chemokine Receptor Binding | 07 | 07/217 | 58/17107 |  | 0.011 |
|  | Platelet Activation | 10 | 10/217 | 143/17107 |  | 0.013 |
|  | CXCR Chemokine Receptor Binding | 04 | 04/217 | 17/17107 |  | 0.020 |
|  | Hemostasis | 14 | 14/217 | 312/17107 |  | 0.020 |
|  | Cytokine Receptor Binding | 13 | 13/217 | 272/17107 |  | 0.020 |
|  | Chemokine Mediated Signaling Pathway | 07 | 07/217 | 73/17107 |  | 0.020 |
|  | Secretory Vesicle | 17 | 17/217 | 462/17107 |  | 0.030 |
|  | Cytokine Activity | 11 | 11/217 | 220/17107 |  | 0.040 |
|  | Cell Junction Organization | 10 | 10/217 | 186/17107 |  | 0.040 |
| M4 | Scavenger Receptor Activity | 06 | 06/164 | 46/17107 |  | 0.005 |
|  | Cargo Receptor Activity | 07 | 07/164 | 67/17107 |  | 0.005 |
| M6 | Regulation of Phosphatidylinositol 3 Kinase Activity | 05 | 05/143 | 41/17107 |  | 0.011 |
|  | Regulation of Phosphatidylinositol 3 Kinase Signaling | 06 | 06/143 | 63/17107 |  | 0.011 |
|  | Positive Regulation of Phosphatidylinositol 3 Kinase Signaling | 06 | 06/143 | 63/17107 |  | 0.011 |
|  | Positive Regulation of Lipid Kinase Activity | 05 | 05/143 | 33/17107 |  | 0.011 |
|  | Positive Regulation of Phospholipid Metabolic Process | 05 | 05/143 | 43/17107 |  | 0.011 |
|  | Regulation of Lipid Kinase Activity | 05 | 05/143 | 49/17107 |  | 0.018 |
|  | Regulation of Phospholipid Metabolic Process | 05 | 05/143 | 62/17107 |  | 0.048 |
| M8 | Lymphocyte Activation | 18 | 18/134 | 343/17107 | < | 0.001 |
|  | Leukocyte Activation | 19 | 19/134 | 415/17107 | < | 0.001 |
|  | Side of Membrane | 19 | 19/134 | 429/17107 | < | 0.001 |
|  | External Side of Plasma Membrane | 13 | 13/134 | 239/17107 | < | 0.001 |
|  | Positive Regulation of Cell Adhesion | 15 | 15/134 | 377/17107 | < | 0.001 |
|  | Lymphocyte Differentiation | 11 | 11/134 | 210/17107 | < | 0.001 |
|  | Positive Regulation of Cell Activation | 13 | 13/134 | 312/17107 | < | 0.001 |
|  | Regulation of Cell Activation | 16 | 16/134 | 485/17107 | < | 0.001 |
|  | Positive Regulation of Cell Cell Adhesion | 11 | 11/134 | 244/17107 | < | 0.001 |
|  | Regulation of Homotypic Cell Cell Adhesion | 12 | 12/134 | 308/17107 | < | 0.001 |
| M9 | Negative Regulation of Epidermis Development | 03 | 03/48 | 17/17107 |  | 0.006 |
|  | Regulation of Hair Follicle Development | 03 | 03/48 | 16/17107 |  | 0.006 |
|  | Regulation of Hair Cycle | 03 | 03/48 | 23/17107 |  | 0.008 |
|  | Regulation of Epidermis Development | 04 | 04/48 | 64/17107 |  | 0.008 |
|  | Benzene Containing Compound Metabolic Process | 03 | 03/48 | 25/17107 |  | 0.009 |
| M13 | Proteinaceous Extracellular Matrix | 06 | 06/37 | 357/17107 |  | 0.009 |
|  | Monocyte Chemotaxis | 03 | 03/37 | 42/17107 |  | 0.009 |
|  | Response to Hydrogen Peroxide | 04 | 04/37 | 110/17107 |  | 0.009 |
|  | Response to Steroid Hormone | 07 | 07/37 | 498/17107 |  | 0.009 |
|  | Glycosaminoglycan Binding | 05 | 05/37 | 206/17107 |  | 0.009 |
|  | Myeloid Leukocyte Migration | 04 | 04/37 | 100/17107 |  | 0.009 |
|  | Taxis | 07 | 07/37 | 465/17107 |  | 0.009 |
|  | Regulation of Lipase Activity | 04 | 04/37 | 84/17107 |  | 0.009 |
|  | Cell Chemotaxis | 05 | 05/37 | 163/17107 |  | 0.009 |
|  | Leukocyte Migration | 06 | 06/37 | 260/17107 |  | 0.009 |

^1^ Only the top 10 significant gene sets are listed.

^2^ The adjustment method was Benjamini-Hochberg.

**Supplementary Table 3. Gene set enrichment analysis of the Early Adversity Group relative to the Control Group**

| Module | NES^1^ | adjusted p-value^2^ | |
| --- | --- | --- | --- |
| M1 | - 1.587 | < | 0.001 |
| M2 | - 1.446 | < | 0.001 |
| M3 | - 1.743 | < | 0.001 |
| M4 | 1.332 |  | 0.009 |
| M5 | 2.701 | < | 0.001 |
| M6 | - 2.431 | < | 0.001 |
| M7 | - 1.236 |  | 0.039 |
| M8 | - 2.508 | < | 0.001 |
| M9 | 2.243 | < | 0.001 |
| M10 | 2.514 | < | 0.001 |
| M11 | 1.562 |  | 0.007 |
| M12 | - 1.466 |  | 0.033 |
| M13 | 2.437 | < | 0.001 |

^1^ NES Normalized Enrichment Score, the enrichment score for a module in each class normalized by the number of genes in the module.
^2^ For each NES, a Benjamini-Hochberg adjusted p-value was calculated.
